# Supplementary material for: Neuropathology and virus in brain of SARS-CoV-2 infected non-human primates
Source: Nat Commun. 2022 Apr 1;13:1745. doi: 10.1038/s41467-022-29440-z (PMC8975902; doi:10.1038/s41467-022-29440-z)
Supplement: Supplementary file 1 — Supplementary Information [file 41467_2022_29440_MOESM1_ESM.pdf]

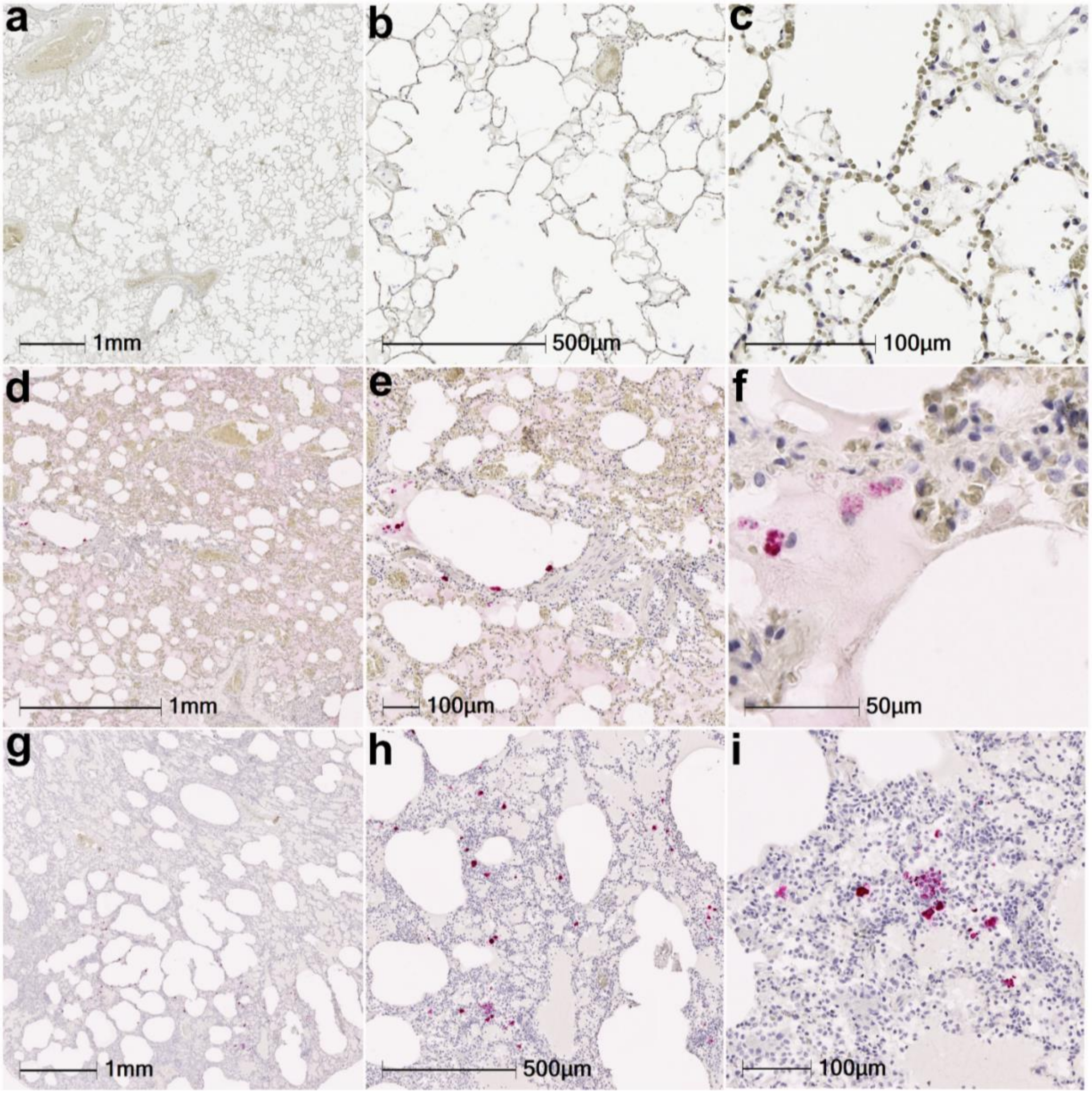

Supplementary Data Figure 1. SARS-CoV-2 in lung. Representative SARS-CoV-2 nucleocapsid protein IHC lung sections from non-infected AGM6 (a-c) and infected NHPs RM2 (d-f) and AGM1 (g-i) are shown at increasing magnification. Viral nucleocapsid was undetectable in lung from mock-infected control animals. In contrast, foci of SARS-CoV-2 nucleocapsid positivity were seen in lung of some infected animals. Immunohistochemical staining for SARS-N in lung was performed thrice. Scale bars = 1 mm (a, d, g), 500 µm (b, h), 100 µm (c, e, i), and 50 µm (f).

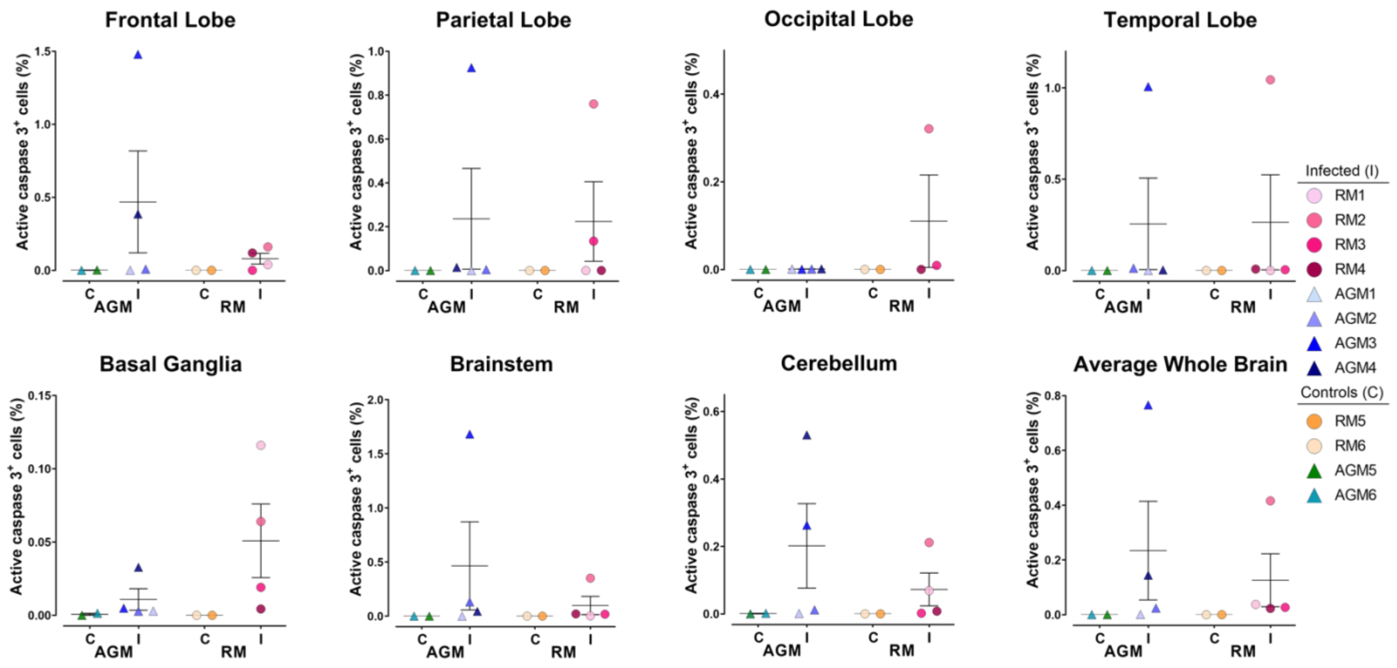

Supplementary Data Figure 2. Cleaved caspase 3 quantitation in brain of SARS-CoV-2 infected NHPs. Graphs show the regional distribution of quantitated caspase 3 positivity in brain that is presented in summation in Figure 2i. Here, n = 2 biologically independent samples/brain region/species in the control groups. Within the infected groups, n = 4 biologically independent samples/brain region/species, except in the occipital lobe, where n = 3 independent samples in the RM infected group. Statistics were performed with a two-tailed Mann-Whitney U test. Data are expressed as mean ± SEM. Source data are provided as a Source Data file. <u>Abbreviations</u>: AGM – African green monkey, RM – Rhesus macaque, C – control, I – infected.

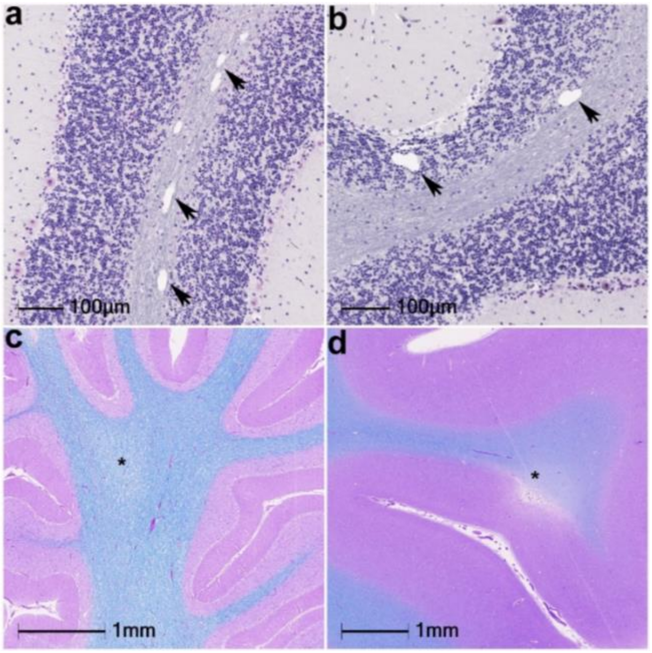

Supplementary Data Figure 3. Structural changes in brain of SARS-CoV-2 infected NHPs. Representative images show areas of vacuolation, indicated by the arrows, within the white matter (a) and granular layer (b) of cerebellum from AGM3, immunoassayed for caspase 3. Representative Luxol Fast Blue images revealed myelin loss, denoted by the asterisks, in cerebellum of RM3 (c) and occipital lobe of AGM3 (d). Luxol fast blue staining was performed once on all brain regions. Scale bars = 100 µm (a, b), 1 mm (c, d).

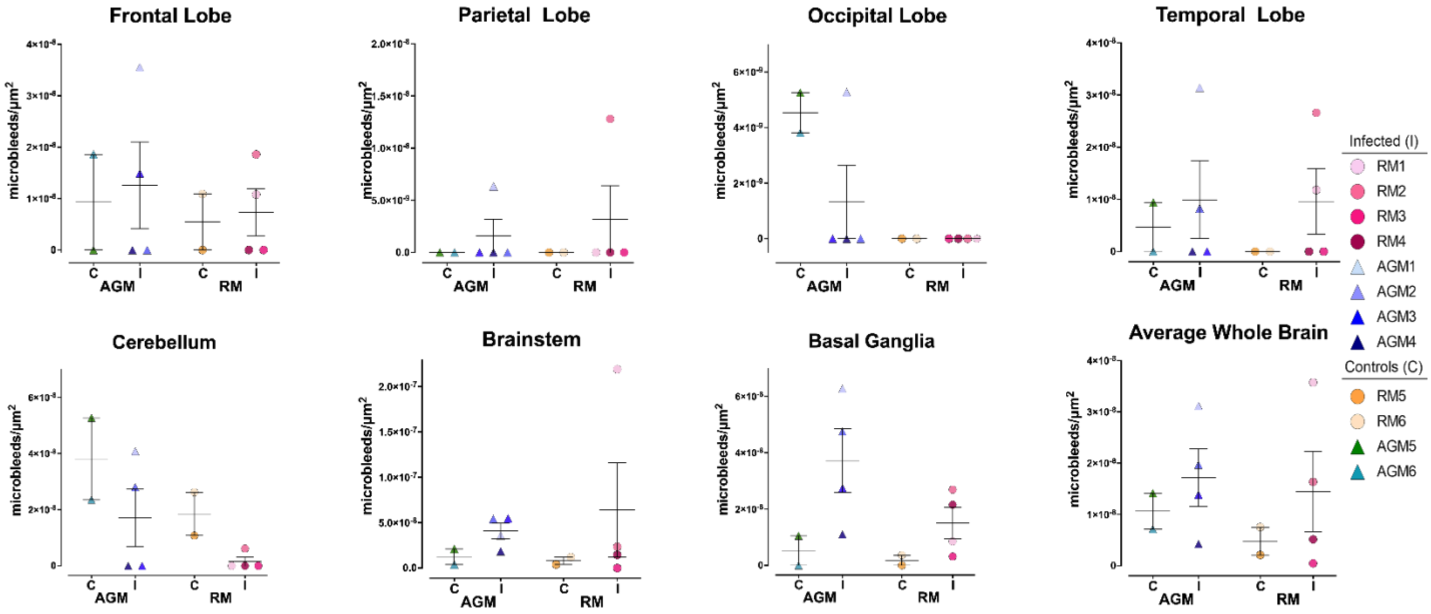

Supplementary Data Figure 4. Regional quantitation of microhemorrhages. Graphs show the number of microbleeds per area (mm^2) stratified by brain region and species from the summated graph in Figure 3g, where n = 2 biologically independent samples/brain region/species in the control groups and n = 4 biologically independent samples/brain region/species in the infected groups. Statistics were performed with a two-tailed Mann-Whitney U test. Data are expressed as mean ± SEM. Source data are provided as a Source Data file. Abbreviations: AGM – African green monkey, RM – Rhesus macaque, C – control, I – infected.

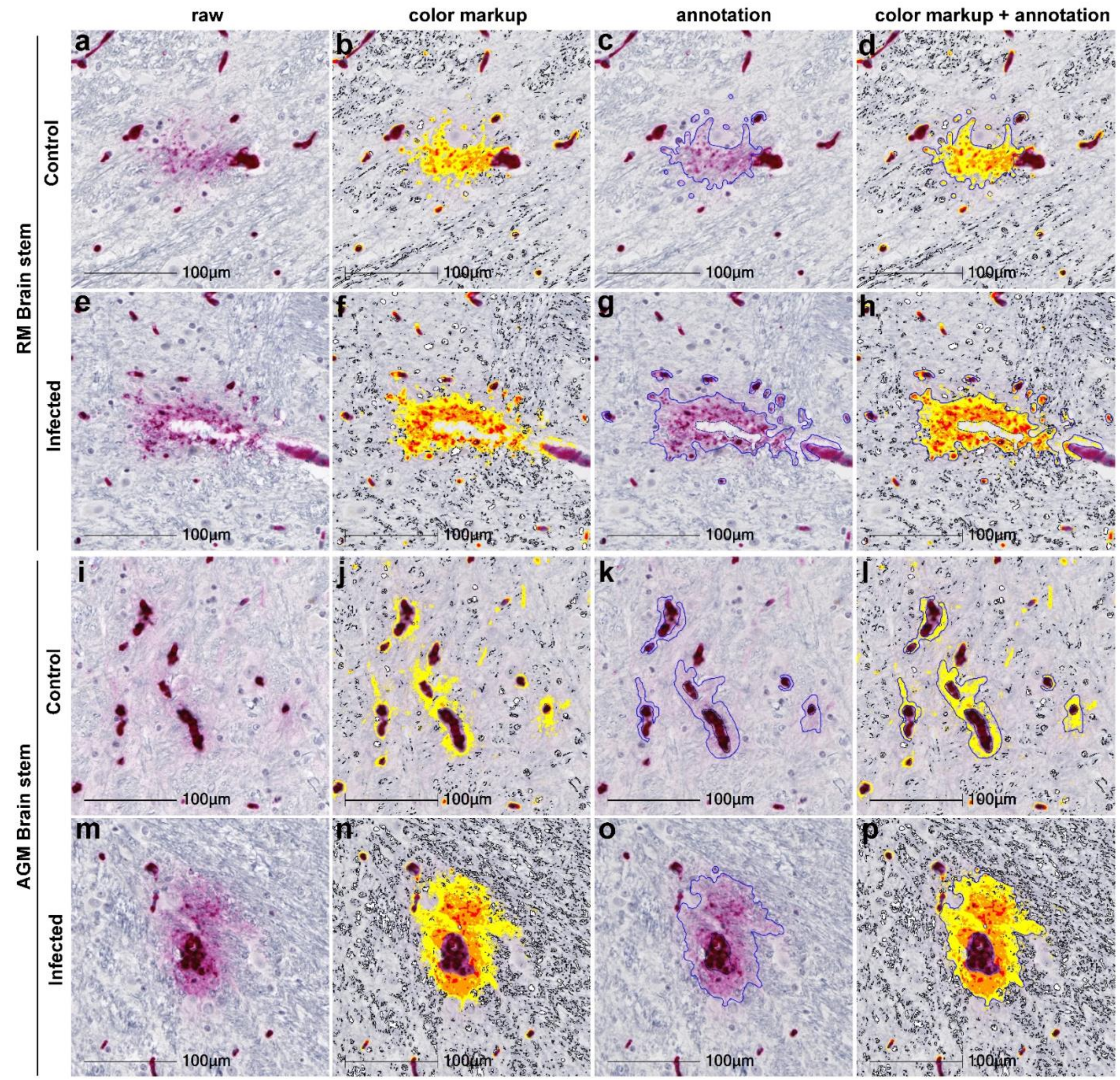

Supplementary Data Figure 5. Quantitation of HIF-1α in brainstem tissues. Example images of the area quantification algorithm markup and the annotations on each tissue section. The raw images for RM5 (a), RM3 (e), AGM5 (i), and AGM4 (m) show blood vessel-associated HIF-1α positivity in parenchyma. Color markup images (b, f, j, n) display the algorithm's assessment for optical density of pixels. Annotations were drawn to outline the markup (c, g, k, o). Solid blue lines indicate regions included in analysis. Dotted blue lines designate areas excluded from analysis within these regions, such as blood vessel lumen, where the deep coloration may obfuscate the dotted line annotation. An overlay ensured precision in these selections (d, h, l, p). <u>Abbreviations</u>: AGM – African green monkey, RM – Rhesus macaque. Scale bars = 100 µm.

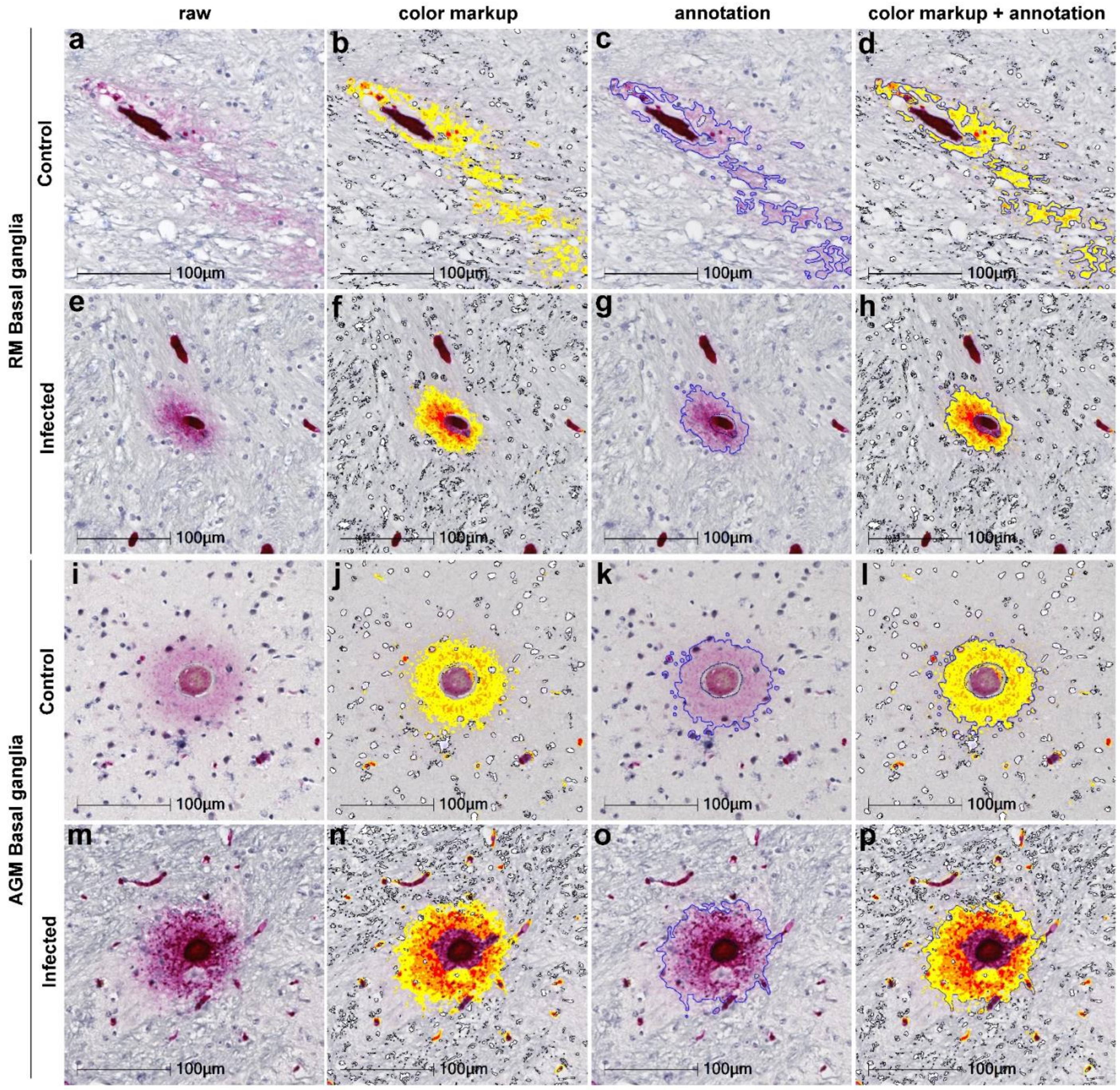

Supplementary Data Figure 6. Quantitation of HIF-1α in basal ganglia. Example images of the area quantification algorithm markup and the annotations on each tissue section. The raw images for RM6 (a), RM3 (e), AGM5 (i), and AGM1 (m) show blood vessel-associated HIF-1α positivity in parenchyma. Color markup images (b, f, j, n) display the algorithm's assessment for optical density of pixels. Annotations were drawn to outline the markup (c, g, k, o). Solid blue lines indicate regions included in analysis. Dotted blue lines designate areas excluded from analysis within these regions, such as blood vessel lumen, where the deep coloration may obfuscate the dotted line annotation. An overlay ensured precision in these selections (d, h, l, p). Abbreviations: AGM – African green monkey, RM – Rhesus macaque. Scale bars = 100 µm.

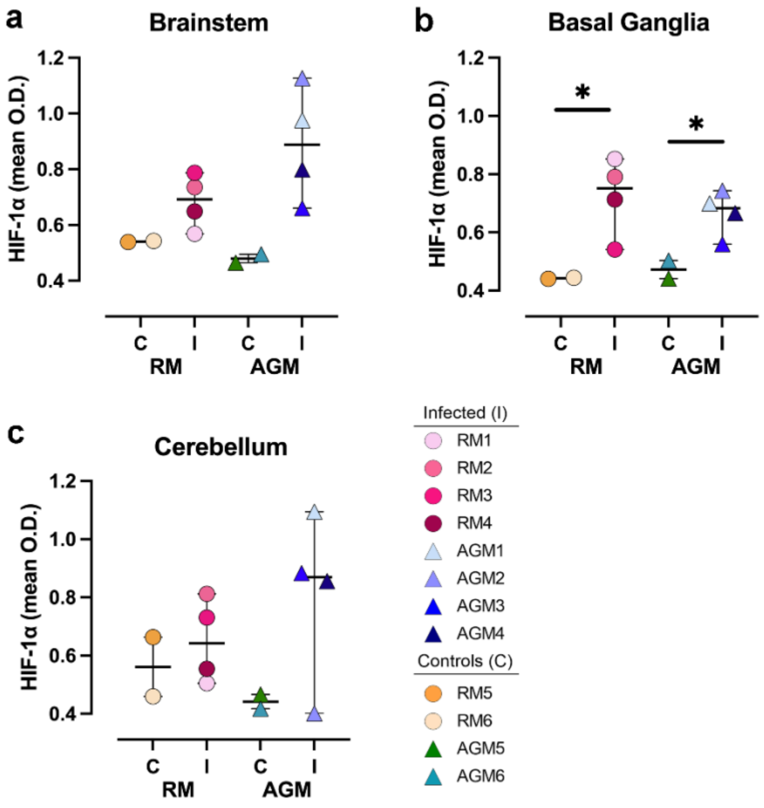

Supplementary Data Figure 7. HIF-1α intensity quantitation stratified by species. When stratified by species, HIF-1α expression is significantly higher in basal ganglia (b) [RM *p = 0.0491 (95%CI = 0.0017 to 0.562) control vs. infected; AGM *p = 0.0344 (95%CI = 0.0233 to 0.3668) control vs. infected]. For statistical analyses, n = 2 biologically independent samples/brain region/species in the control groups and n = 4 biologically independent samples/brain region/species in the infected groups. Although mean HIF-1α is greater in the context of infection for both species, this does not reach statistical significance in brainstem (a) or cerebellum (c). Statistics were done with unpaired two-tailed t test. Data are expressed as mean ± SEM. Source data are provided as a Source Data file. Abbreviations: AGM – African green monkey, RM – Rhesus macaque, C – control, I – infected, O.D. – optical density.

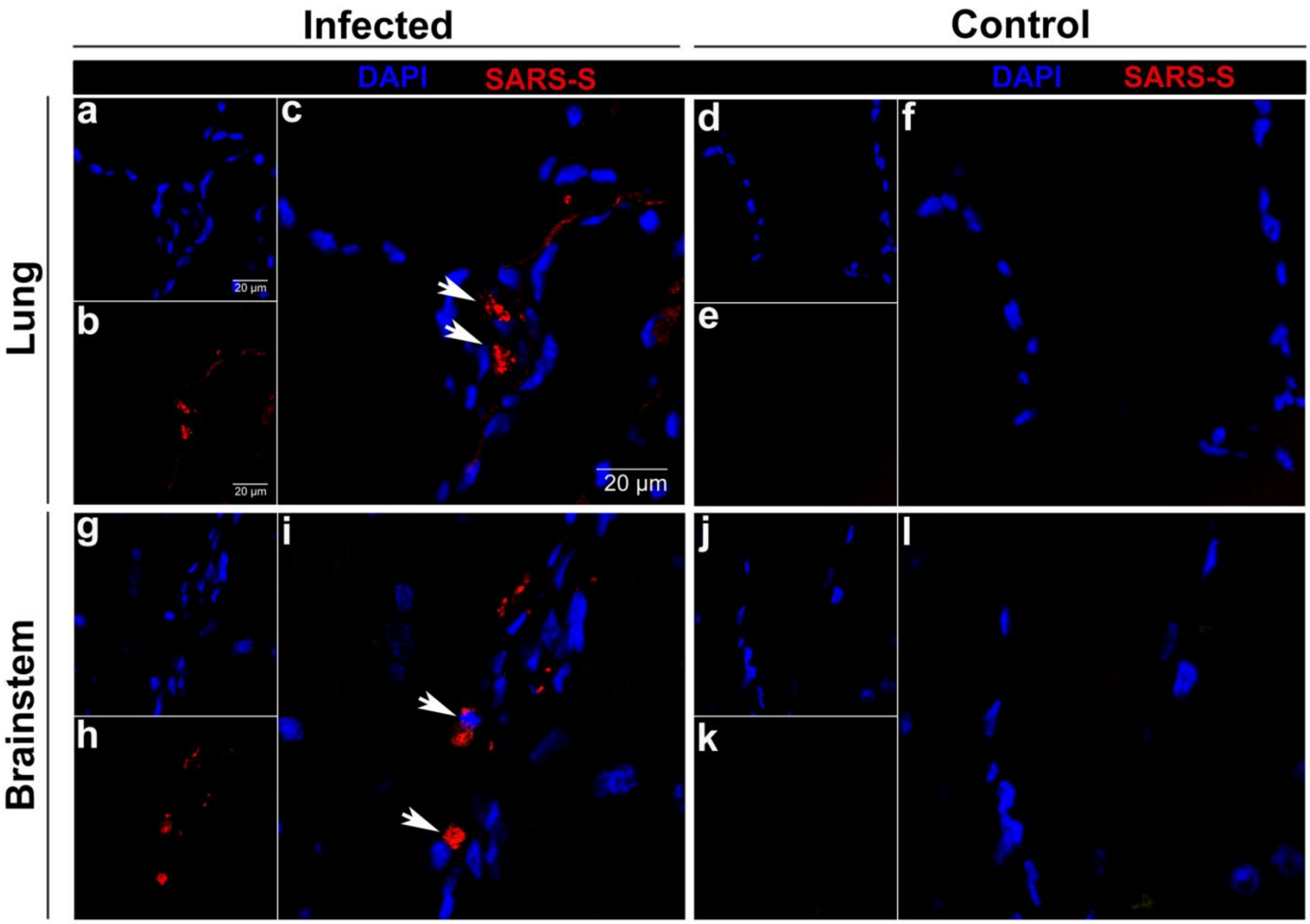

Supplementary Data Figure 8. SARS-CoV-2 Spike RNA in lung and brain. Representative images of RNAscope (in situ hybridization) detection of SARS-CoV-2 spike RNA (SARS-S) in lung AGM5 and brainstem AGM6 of control animals (d-f; j-l) and infected AGM1 and RM1 (a-c; g-i), respectively. Cytoplasmic SARS-CoV-2 spike RNA positivity, indicated by red color, was only rarely observed in infected tissues which is shown in AGM1 and RM1. Control tissues showed no signs of positivity. Blue color represents DAPI labelled cell nuclei. Scale bars = 20 µm.
