# Supplementary material for: Neuropathology and virus in brain of SARS-CoV-2 infected non-human primates
Source: Nat Commun. 2022 Apr 1;13:1745. doi: 10.1038/s41467-022-29440-z (PMC8975902; doi:10.1038/s41467-022-29440-z)
Supplement: Supplementary file 3 — Reporting Summary [file 41467_2022_29440_MOESM3_ESM.pdf]

nature research

Corresponding author(s): Tracy Fischer

Last updated by author(s): Mar 1, 2022

Reporting Summary

Nature Research wishes to improve the reproducibility of the work that we publish. This form provides structure for consistency and transparency in reporting. For further information on Nature Research policies, see our Editorial Policies and the Editorial Policy Checklist.

Statistics

For all statistical analyses, confirm that the following items are present in the figure legend, table legend, main text, or Methods section.

n/a	Confirmed	
☐	☒	The exact sample size (n) for each experimental group/condition, given as a discrete number and unit of measurement
☐	☒	A statement on whether measurements were taken from distinct samples or whether the same sample was measured repeatedly
☐	☒	The statistical test(s) used AND whether they are one- or two-sided<br>*Only common tests should be described solely by name; describe more complex techniques in the Methods section.*
☒	☐	A description of all covariates tested
☐	☒	A description of any assumptions or corrections, such as tests of normality and adjustment for multiple comparisons
☐	☒	A full description of the statistical parameters including central tendency (e.g. means) or other basic estimates (e.g. regression coefficient) AND variation (e.g. standard deviation) or associated estimates of uncertainty (e.g. confidence intervals)
☐	☒	For null hypothesis testing, the test statistic (e.g. F, t, r) with confidence intervals, effect sizes, degrees of freedom and P value noted<br>*Give P values as exact values whenever suitable.*
☒	☐	For Bayesian analysis, information on the choice of priors and Markov chain Monte Carlo settings
☒	☐	For hierarchical and complex designs, identification of the appropriate level for tests and full reporting of outcomes
☒	☐	Estimates of effect sizes (e.g. Cohen's d, Pearson's r), indicating how they were calculated

Our web collection on statistics for biologists contains articles on many of the points above.

Software and code

Policy information about availability of computer code

Data collection	Axio Scan.Z1 digital slide scanner (Ziess), Leica DMi8 automated confocal microscope, model SP8 (Leica), Olympus IX73 inverted microscope (Olympus), and HALO (Indica Labs, v2.3.2089. 70 and v3.1.1076.405) were used to obtain bright field and fluorescent images.
Data analysis	HALO (Indica Labs, v2.3.2089. 70 and v3.1.1076.405) was used for threshold and multiplex analyses of caspase 3 and the optical density measurement of HIF-1a. GraphPad Prism software v9.0.2. was used for statistical analysis. Leica imaging software suite LAS X (Leica, v3.5.7.23225) and cellSens Dimension 3 (Olympus, v3.1) were used to acquire fluorescent images. Photoshop (Adobe, v21.2.0) was used to adjust brightness, contrast, and darken midtones uniformly, then overlay these images.

For manuscripts utilizing custom algorithms or software that are central to the research but not yet described in published literature, software must be made available to editors and reviewers. We strongly encourage code deposition in a community repository (e.g. GitHub). See the Nature Research guidelines for submitting code & software for further information.

Data

Policy information about availability of data

All manuscripts must include a data availability statement. This statement should provide the following information, where applicable:

- Accession codes, unique identifiers, or web links for publicly available datasets
- A list of figures that have associated raw data
- A description of any restrictions on data availability

A data source file has been made publicly available through Figshare. The file can be accessed through this link provided: https://figshare.com/articles/dataset/COVID_NHP_CNS_Source_Data/19241727
Figures with associated raw data include figures 2i; 3g, h; 5c-e; 6o and extended data figures 2; 4; 7a-c.

Field-specific reporting

Please select the one below that is the best fit for your research. If you are not sure, read the appropriate sections before making your selection.

☒ Life sciences ☐ Behavioural & social sciences ☐ Ecological, evolutionary & environmental sciences

For a reference copy of the document with all sections, see nature.com/documents/nr-reporting-summary-flat.pdf

Life sciences study design

All studies must disclose on these points even when the disclosure is negative.

Sample size	The animal models are new and no prior data existed at the time of initiation of our studies. Our objective was to identify and characterize probable neuropathology of infected animals. Based on our expertise on the impact of peripheral infection on neuroinflammation in humans and non-human primates, we hypothesized that infected animals would experience greater neuroinflammation, as indicated by expression of specific antigens of interest, morphological changes by reactive glia, and nodular lesions, as compared to age-matched mock-infected animals, with a large effect size greater than or equal to 85% (1-3).<br>References:<br>1. Tavazzi E, Morrison D, Sullivan P, Morgello S, Fischer T. Brain inflammation is a common feature of HIV-infected patients without HIV encephalitis or productive brain infection. Curr HIV Res 12, 97-110 (2014).<br>2. Gerngross L, Fischer T. Evidence for cFMS signaling in HIV production by brain macrophages and microglia. J Neurovirol 21, 249-256 (2015).<br>3. Gerngross L, Lehmicke G, Belkadi A, Fischer T. Role for cFMS in maintaining alternative macrophage polarization in SIV infection: implications for HIV neuropathogenesis. J Neuroinflammation 12, 58 (2015).
Data exclusions	No data were excluded.
Replication	Detailed experimental design is provided within the manuscript, including the age, sex, inoculation strategy and virus exposure, study length, and nasal swab viral load. After euthanasia, brain was removed within 2 hours and frozen or placed in zinc formalin (Z-Fix) for 72 hours. All tissues were processed similarly by the Histopathology Core within the TNPRC.<br>Immunohistochemistry and immunofluorescence studies were performed using previously titrated antibodies. All assays were performed on the same brain region for each study animal within the same run to ensure rigor and reproducibility. Similarly, special stains were performed on brain tissues from all animals at the same time, using the same solutions. One 5 um thick section from each animal was randomly selected for IHC and IF. Each assay was performed at least twice on all brain regions, with the exception of SARS-CoV-2 nucleocapsid IHC/IF, which was performed 12 times. RNAscope analysis for SARS-CoV-2 spike protein was performed 7 times. The inclusion of positive control tissues verified that all runs were performed successfully. Special stains were successfully performed as follows: H&E was completed 4 times, Luxol fast blue once, and FluoroJade C twice. Slides were evaluated and scored by bright field microscopy, scanned (AxioScan), and analyzed using HALO. Caspase 3 and HIF-1a quantitation was performed on entire raw brain images. IHC images displayed were adjusted for brightness and contrast, which was applied to the entire image.
Randomization	Animals were randomly assigned to one of the two inoculation strategy groups.
Blinding	Blinding was used for in-lab scoring of pathology and intensity/expression level of antigen (interpretation at microscopic viewing). Blinding was not necessary for computerized HALO analyses, as the same non-biased quantitation conditions were applied to all samples for each immunostaining.

Reporting for specific materials, systems and methods

We require information from authors about some types of materials, experimental systems and methods used in many studies. Here, indicate whether each material, system or method listed is relevant to your study. If you are not sure if a list item applies to your research, read the appropriate section before selecting a response.

Materials & experimental systems

n/a	Involved in the study	
☐	☒	Antibodies
☒	☐	Eukaryotic cell lines
☒	☐	Palaeontology and archaeology
☐	☒	Animals and other organisms
☒	☐	Human research participants
☒	☐	Clinical data
☒	☐	Dual use research of concern

Methods

n/a	Involved in the study	
☒	☐	ChIP-seq
☒	☐	Flow cytometry
☒	☐	MRI-based neuroimaging

Antibodies

Antibodies used	Primary antibodies included: anti-cleaved caspase 3 (Abcam, ab2302); anti-von Willebrand Factor (EPR12010, Abcam, ab179451); anti-HIF-1a (Abcam, ab16066); anti-CD61 (Invitrogen, MA5-33041); anti-Iba1 (Abcam, ab5076), anti-GFAP (Abcam, ab68428); anti-

HLA-DR (Novus, NB600989); and anti-SARS-CoV-2 nucleocapsid (Novus, NB100-56576). Secondary antibodies included: biotinylated horse anti-rabbit IgG (Vector Labs, BA-1100), biotinylated horse anti-mouse IgG (Vector Labs, BA-2000), biotinylated horse anti-goat IgG (Vector Labs, BA-9500), Alexa-Fluor 488 goat anti-rabbit IgG (Invitrogen, A11008), and Alexa-Fluor 555 goat anti-rabbit IgG (Invitrogen, A21428)

Validation

Validation of primary antibodies used in immunohistochemistry studies is titrated on FFPE tissue, which is then included as a positive control for subsequent investigative studies. Validation/positive control tissues are selected based on vendor suggestions and/or literature. A range of dilutions are included in the titration studies for optimization. Afterwards, if brain is not used for validation for a specific antibody, additional validation and titration of the antibody is performed on brain. Test runs are performed on select brain regions from infected and mock-infected control animals simultaneously to account for differences between infection state. To ensure rigor and reproducibility, each run was performed on individual brain regions from all animals at the same time and using the same solutions.

Anti-cleaved caspase 3 was titrated on tonsil and brain from infected animals (AGM), anti-von Willebrand Factor was titrated on brain from non-infected animals (RM), anti-HIF-1a was titrated on large intestine and brain of infected and non-infected animals (RM and AGM), anti-CD61 was titrated on spleen and brain of infected animals (AGM), anti-Iba1 was titrated on brain of infected and non-infected animals (RM and AGM), anti-GFAP was titrated on brain of infected and non-infected animals (RM and AGM), anti-HLA-DR was titrated on brain of infected and non-infected animals (RM and AGM), anti-SARS-CoV-2 nucleocapsid was titrated on lung and brain from infected animals (AGM).

Animals and other organisms

Policy information about studies involving animals; ARRIVE guidelines recommended for reporting animal research

Laboratory animals

Six Indian-origin Rhesus macaques (RMs; ages 13-21 years; 2 female, 4 male) were acquired from the Tulane National Primate Research Center specific pathogen-free breeding colony and confirmed negative for simian type D retrovirus (SRV), simian immunodeficiency virus (SIV), simian T cell lymphotropic/leukemia virus (STLV), measles virus (MV), and tuberculosis (TB).

Wild animals

Six wild-caught African green monkeys (AGMs) of Caribbean origin (all approximately 16 years of age; 3 female, 3 male). AGMs were captured in baited cages from the island of Saint Kitts. Animals were transported in cages approved by the International Air Transport Association by direct charter flight to the US and quarantined according to CDC guidelines prior to transport to Tulane. Animals were housed at the Tulane National Primate Research Center greater than one year before assignment to this study. The AGMs were confirmed negative for SRV, SIV, STLV, MV, and TB. Tulane has a written endpoint policy to minimize potential pain and distress experienced by animals. The policy addresses limits on weight loss, appetite, tumor size (if present), response to medical intervention, activity, and a number of other clinical signs relevant to laboratory animal species. The Tulane endpoint policy requires that animals be euthanized or removed from study when endpoints are reached. As per the study protocol, animals were euthanized at the end of the defined study period unless humane endpoints were reached prior. When euthanasia is indicated, buprenorphine and tiletamine/zolazepam are administered intramuscularly to induce a deep plane of anesthesia and analgesia. Euthanasia is then performed by intracardiac injection of sodium pentobarbital.

Field-collected samples

No samples were collected at the field.

Ethics oversight

All animal studies were approved by the Tulane University Institutional Animal Care and Use Committee (IACUC) and carried out in the Regional Biocontainment Laboratory at the Tulane National Primate Research Center (TNPRC) within an animal biosafety level 3 (ABSL3) facility. TNPRC is an AAALAC accredited facility. All animals were cared for in accordance with the Institute for Laboratory Animal Research (ILAR) Guide for the Care and Use of Laboratory Animals, 8th edition. The Tulane University Institutional Biosafety Committee (IBC) approved all procedures for sample handling, inactivation, and removal from BSL3 containment.

Note that full information on the approval of the study protocol must also be provided in the manuscript.
